# Hypertrophic Nonunion Humerus Mimicking an Enchondroma

**DOI:** 10.1155/2014/854349

**Published:** 2014-11-30

**Authors:** N. K. Magu, Amanpreet Singh, Reetadyuti Mukhopadhyay, Jitendra Wadhwani, Paritosh Gogna, Rohit Singla, Sahil Arora, Pragnashree Mukhopadhyay Chatterjee

**Affiliations:** ^1^Department of Orthopaedics, Pt. B.D. Sharma PGIMS, Rohtak 124001, India; ^2^Hinduja Sindhi Hospital, Bangalore, Karnataka 560027, India

## Abstract

*Introduction*. Although fractures of humeral shaft show excellent results with conservative management, nonunion does occur.* Case Report*. We bring forth the case of a young male with a 1.5-year-old hypertrophic nonunion of the humerus mimicking an enchondroma. The initial X-ray images of the patient appeared to be an enchondroma, which only on further evaluation and histopathological analysis was diagnosed conclusively to be a hypertrophic nonunion.* Discussion*. Enchondromas are often incidentally diagnosed benign tumours. It is however not common to misdiagnose a hypertrophic nonunion to be an enchondroma. We present this case to highlight the unique diagnostic dilemma the treating team had to face.

## 1. Introduction

Three percent of all fractures occur in the humeral shaft. Fracture shaft of humerus shows excellent results with nonoperative treatment in cast immobilization and bracing [1, 2]. However, nonunion is known to occur, particularly in our setting where patients seek treatment from traditional bonesetter [3]. Results following surgical management are variable depending upon the choice of fixation method and implant used, each having its own set of complications and morbidities [4]. When nonunion does occur, however, it is very difficult to treat and often requires multiple procedures to achieve union. Even with multiple procedures, true pseudoarthroses have only a 59% union rate [5].

## 2. Case Report

In this report we present an interesting case of hypertrophic nonunion of humeral shaft fracture in a 14-year-old boy. In the first instance the X-ray picture appeared to mimic an enchondroma.

He was a fully independent, medically fit and well, right-handed, and a nonsmoker who presented to the outpatient department with a deformity of the left arm. He had history of a minor fall about 1.5 years back followed by pain in left arm. He had visited a local bonesetter who gave him a massage and local bandage. The bandage was removed after 3 weeks. The pain had settled; however the patient was unable to lift weight with his left upper limb and had complained of increasing deformity in the proximal arm. He also complained of difficulty to carry out daily activities with his left upper limb.

On examination, he had a good range of motion in his shoulder and had an intact neurovascular status. While examining his arm, a completely asymptomatic mass was readily palpable over the middle left humerus with a detectable motion in the midhumerus.

**Figure 1:**
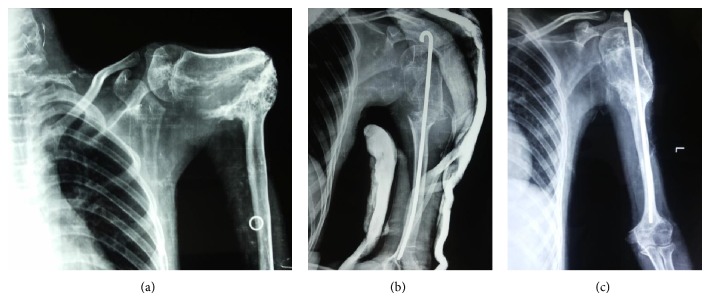
(a) Preoperative X-ray showing the lesion and the degree of deformity which at first appeared to the treating team to be an enchondroma. (b) Immediate postoperative X-rays in U slab. (c) X-rays at final follow-up showing union.

**Figure 2:**
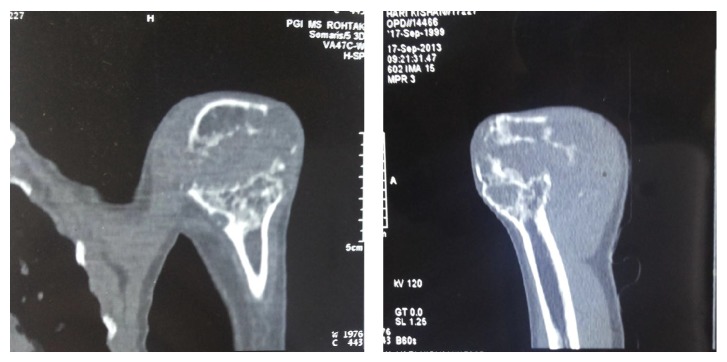
Preoperative CT showing exuberant new bone formation at the site of the deformity.

Plain radiographs demonstrated a suspicious solitary, diaphyseal humeral lesion, sparing the cortices, and surrounding soft tissues with an abundance of bone formation (Figure 1(a)). The CT revealed a lesion with exuberant bone formation at the site of deformity (Figure 2).

The patient was planned for surgery after preanesthetic checkup. Intraoperatively the site was exposed and a sample was collected for histopathological examination. The excess callus was trimmed, and after freshening of the lesion site and clearing of the marrow, an intramedullary Rush nail was passed with an entry point at the lesser tuberosity (Figure 1(b)). Postoperatively the patient had an intact neurovascular status.

**Figure 3:**
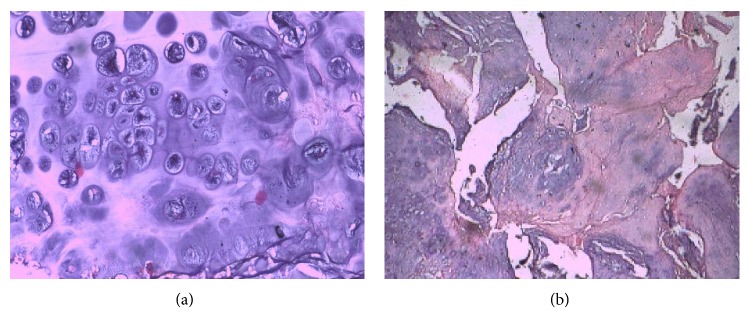
Histopathology showing features suggestive of callus formation with no evidence of malignancy.

The histopathology report was suggestive of callus, with features suggestive of osteoid formation, with no features of malignancy (Figure 3).

The patient was given a U slab postoperatively for 14 days; on the 14th postoperative day the slab was removed and sutures were cut. The wound was healthy with no suture line redness or discharge. A shoulder spica was applied for 6 weeks thereafter. The plain radiograph taken after 8 weeks of surgery showed features of union. At the end of 8 weeks postoperatively the spica was removed and the patient was advised careful physiotherapy of the elbow and shoulder.

**Figure 4:**
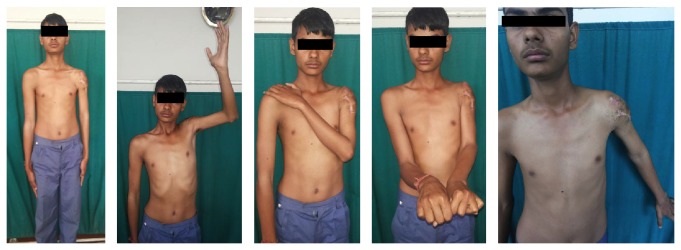
Patient at final follow-up showing good range of motion.

With regular follow-up and physiotherapy, at the end of 5 months of follow-up, the patient had a completely united fracture (Figure 1(c)) with good range of motion at the shoulder and the elbow (Figure 4).

## 3. Discussion

Enchondromas are often diagnosed incidentally based on radiographic appearance. They are the second most common benign tumours of bone and make about one-fifth of all cartilage forming tumours. They present as either a monostotic or polyostotic form. The solitary forms have a very low risk of malignant transformation, while the ones associated with enchondromatosis show close to 25% malignant transformation. They typically present between the second and fourth decade, located centrally within the marrow of the bone, with a predilection for short tubular bones, followed by the proximal humerus and femur. The enchondromas are often asymptomatic tumours [6].

Most humeral diaphyseal fractures heal with conservative management [7]. However, when nonunion occurs, it is probably due to the severity of the initial trauma, the pattern of the fracture, distraction at the fracture site, soft-tissue interposition, or inadequate immobilisation [8]. There are various surgical methods to treat nonunion after conservative management. The various options include open reduction and internal fixation with plates and screws [9], reamed intramedullary nailing [10], and external fixation [11]. Supplementing fixation with bone graft significantly increases the chances of achieving union [12].

Our case was unique in presenting eighteen months of the initial trauma with a deformity of almost 90° at the fracture site, which on initial X-rays mimicked an enchondroma. It was only on further investigation and histopathological analysis that it was conclusively diagnosed to be a hypertrophic nonunion with exuberant callus formation. The fracture finally united without grafting with intramedullary fixation supplemented by a spica cast.

We report this case to bring forth the diagnostic dilemma the initial X-rays resembling an enchondroma brought in front of the treating team.
